# Supplementary figures and images for: Multiple Viral microRNAs Regulate Interferon Release and Signaling Early during Infection with Epstein-Barr Virus
Source: mBio. 2021 Mar 30;12(2):e03440-20. doi: 10.1128/mBio.03440-20 (PMC8092300; doi:10.1128/mBio.03440-20)

**A**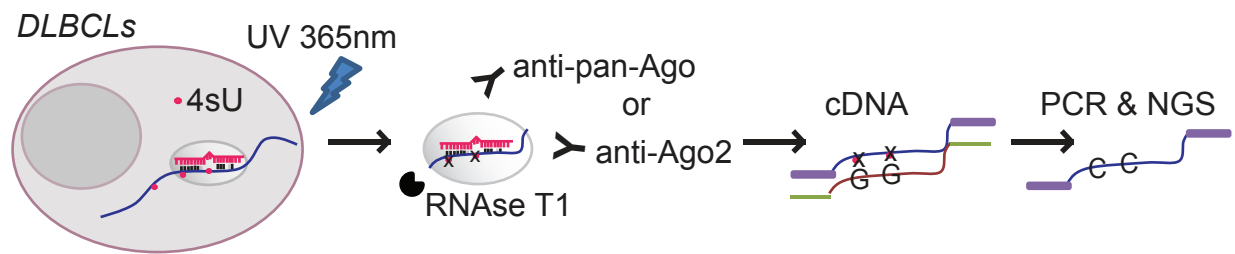**B**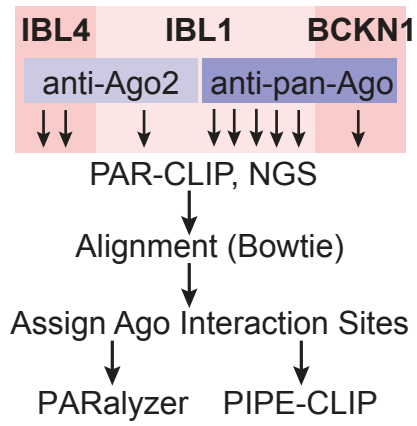**C**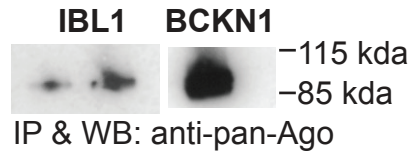**D**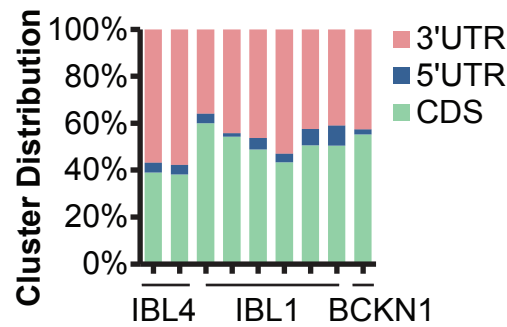**E**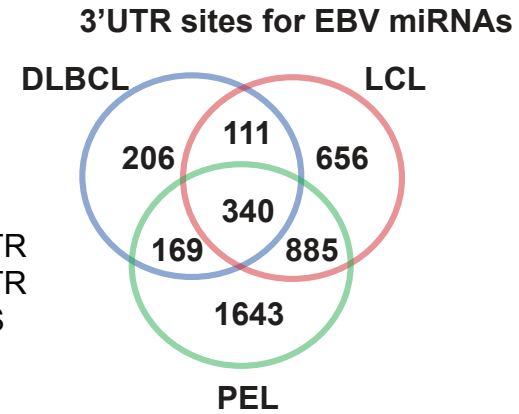**F**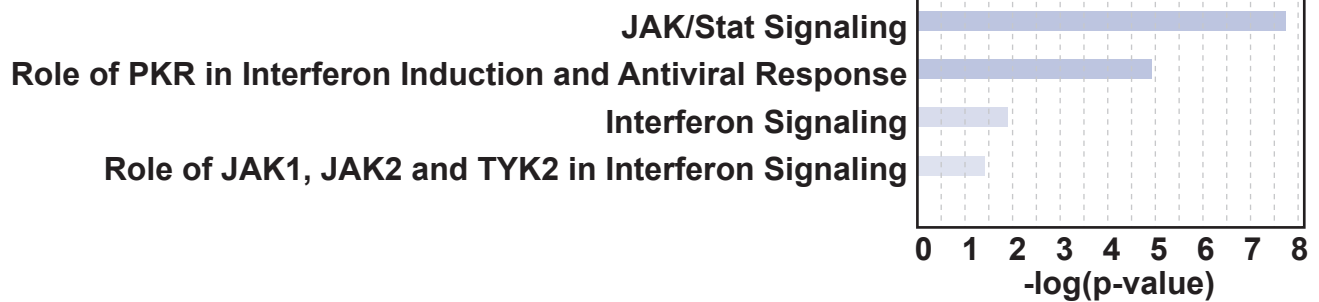

**Supplementary Figure 1**

Supplement: FIG S1 [file mBio.03440-20-sf001.pdf]

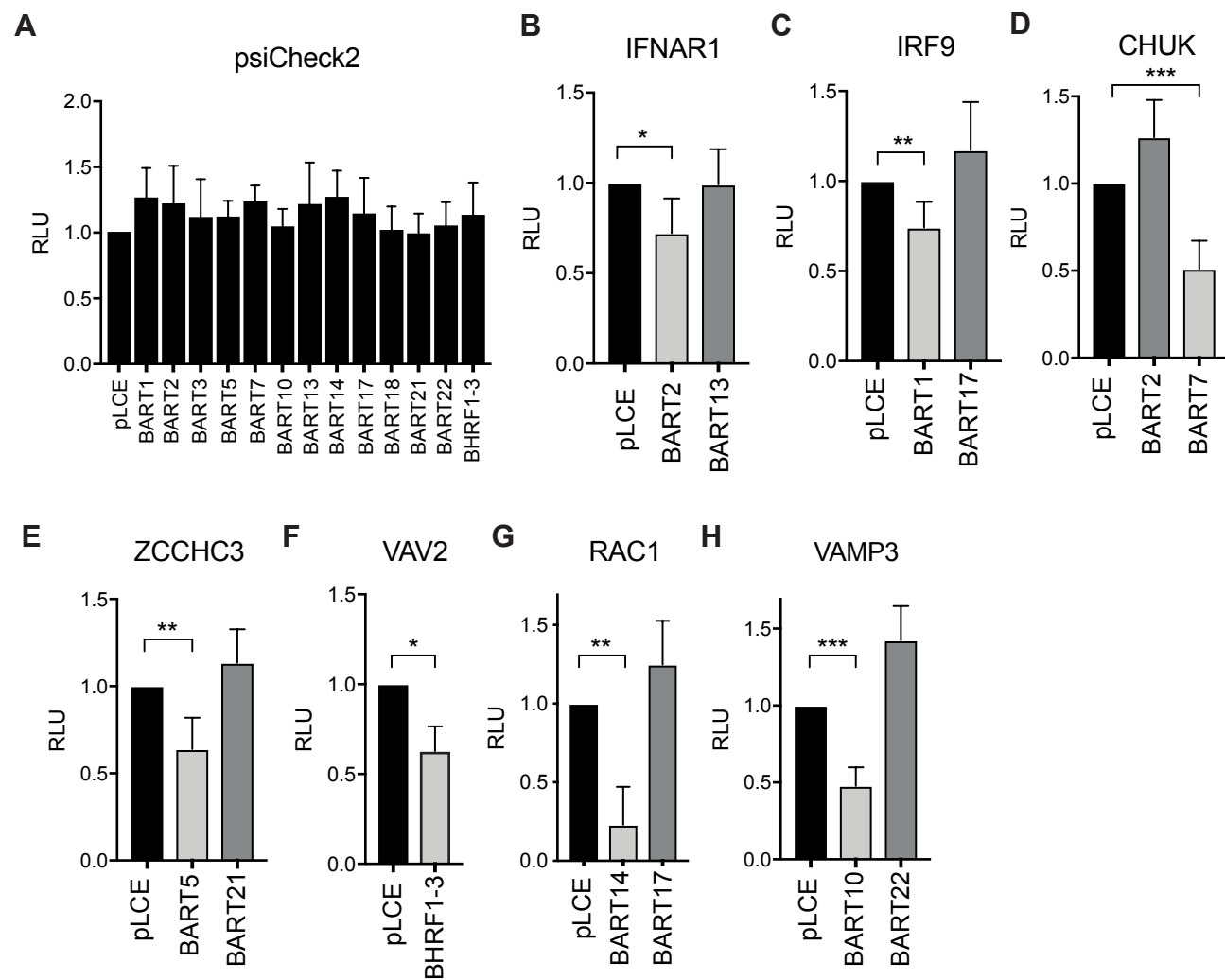

Supplementary Figure 3

Supplement: FIG S3 [file mBio.03440-20-sf003.pdf]

**A**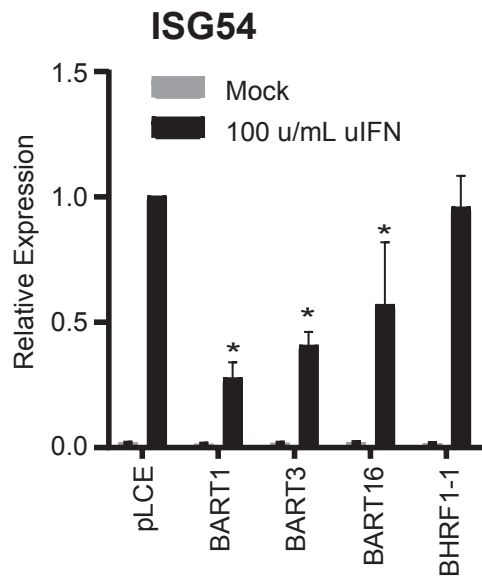**B**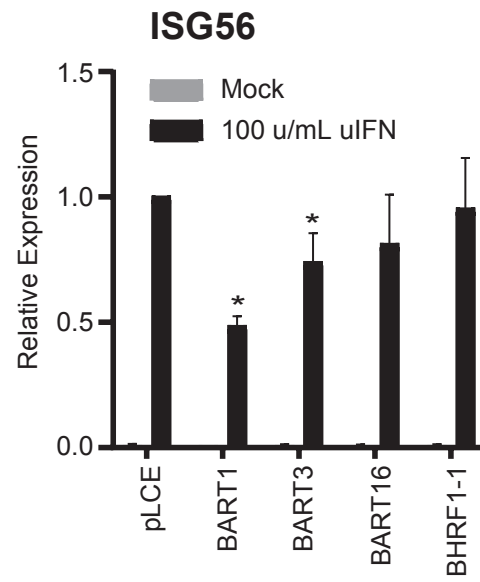

Supplement: FIG S4 [file mBio.03440-20-sf004.pdf]

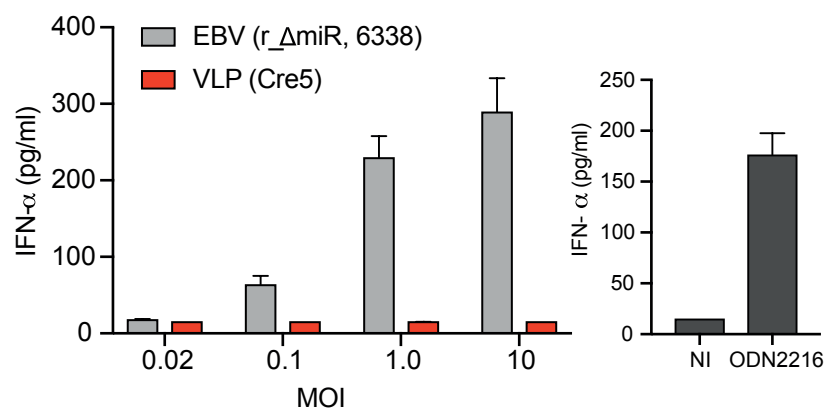

**Supplementary Figure 5**

Supplement: FIG S5 [file mBio.03440-20-sf005.pdf]

**B cells**

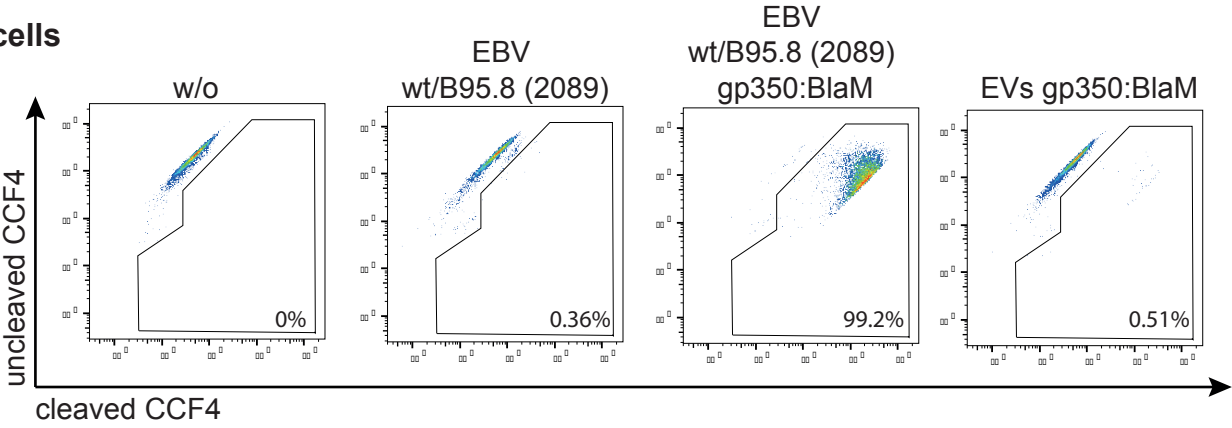

**pDCs**

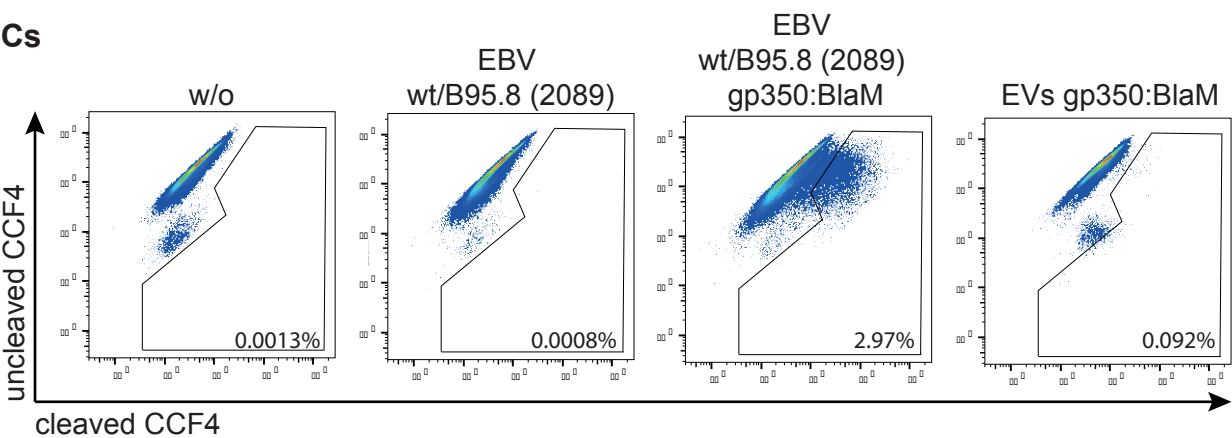

**Supplementary Figure 6**

Supplement: FIG S6 [file mBio.03440-20-sf006.pdf]

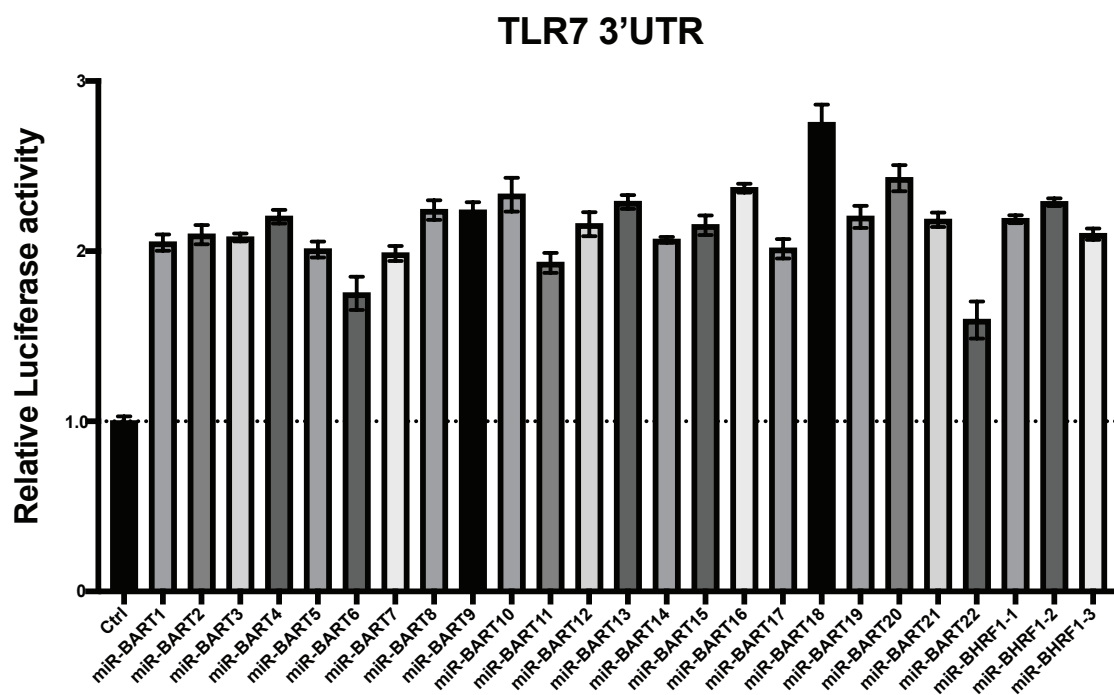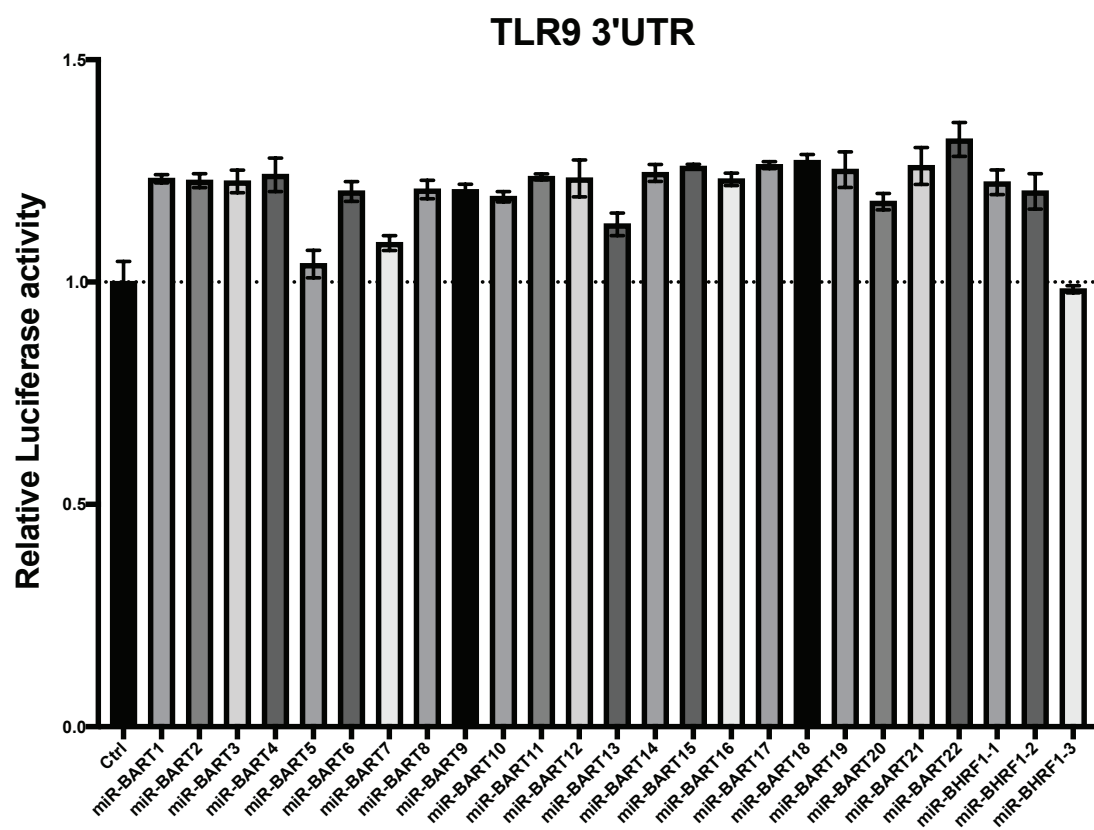

Supplementary Figure 7

Supplement: FIG S7 [file mBio.03440-20-sf007.pdf]
