# Supplementary material for: Multiple Viral microRNAs Regulate Interferon Release and Signaling Early during Infection with Epstein-Barr Virus
Source: mBio. 2021 Mar 30;12(2):e03440-20. doi: 10.1128/mBio.03440-20 (PMC8092300; doi:10.1128/mBio.03440-20)
Supplement: FIG S2 [file mBio.03440-20-sf002.pdf]

RIG-I 3'UTR

mut miR-BART3

```
5'CAUGCAAUCUGGUGAGCAGUGGUGCAGGC 3'
                     ||||||||
      3' UGUGGACCACUGAUCACCACGC 5'  miR-BART3
                     ||****||
5'  CAUGCAAUCUGGUGAGCAGACCAGCAGGC 3'
```

mut miR-BART19-5p

```
5' CCUCAAUCUUCAGCUACAGGGAAUGAGUA 3'
                     |||||||
   3' GUACAGUACAAACGCCCCUUACA 5'  miR-BART19-5p
                     ||****|
5' CCUCAAUCUUCAGCUACAGGCUUAGAGUA 3'
```

Viperin 3'UTR

mut miR-BART9

```
5'UACAGCAGGUAUUCAAUCAGUGUUAUUUG 3'
                      |||||||
  3' UGAUGCCCUGGGUACUUCACAAU 5'  miR-BART9
                      |****||
5'  UACAGCAGGUAUUCAAUCACACAUAUUUG 3'
```

mut miR-BART19-5p

```
5'AGUUUGAAUCUUCUGAGUUGGAAUGAAUU 3'
                      ||||||
    3' GUACAGUACAAACGCCCCUUACA 5'  miR-BART19-5p
                      |****|
5'  AGUUUGAAUCUUCUGAGUUGCUUAGAAUU 3'
```

mut miR-BART21-3p

```
5' AAAAAAAAGCAAGAGAGUUCAACUAAGAA 3'
                      ||||||
     3' UUUGUGGUCACCCGUGUUGAUC 5'  miR-BART21-3p
                      ****||
5' AAAAAAAAGCAAGAGAGUUGUUGUAAGAA 3'
```

IKKβ 3'UTR

mut miR-BART17-5p

```
5' CCUGGCCCCAUCCUCACUUCCUCUUUUUA 3'
                     |||||||
     3' GAACAUACGGACGCAGGAGAAU 5'  miR-BART17-5p
                     ||****|
5' CCUGGCCCCAUCCUCACUUCGAGAUUUUA 3'
```

IRAK2 3'UTR

mut miR-BART22

```
5' UUUUGGGACCUCAGUUUCUUUGUAAGUAA 3'
                     ||||||||
   3' UGAUGAUCUGGUACUGAAACAUU 5'  miR-BART22
                     ||****||
5' UUUUGGGACCUCAGUUUCUAACAAAGUAA 3'
```

Fyn 3'UTR

mut miR-BART10

```
5' AAAUGCAUUGUAUCGAUGUUAUGUAAAAG 3'
                     |||| ||||
   3' UGUCGGUUGAGGUACCAAUACAU 5'  miR-BART10
                     ||****||
5' AAAUGCAUUGUAUCGAUGUAUACUAAAAG 3'
```

Supplementary Figure 2

CBP 3'UTR

mut miR-BART16

```
5'ACACACGUACACACCCACACACAUCUAUCUAUACAUAAU 3'
                        ||||||||
    3' UCUCGUGUGUGGGUGAGAUAGAUU 5'  miR-BART16
                        ||****||
5'  ACACACGUACACACCCACACACAUCAUAGUAUACAUAAU 3'
```

IRF9 3'UTR

mut miR-BART1-5p

```
5'UAUAUUUUCCUCUUAGAUAUUCACUAAGGACUUA 3'
                       |||||||
 3' GUGUCGUGCAGUGAAGGUGAUUCU 5'  miR-BART1-5p
                       |****||
5'  UAUAUUUUCCUCUUAGAUAUUCUGAUAGGACUUA 3'
```

JAK1 3'UTR

mut miR-BART3-3p

```
5' UUUACCAUCAAUCAUCACCCUGUGGUGCAACACUU 3'
                         |||||||
   3' UGUGGACCACUGAUCACCACGC 5'  miR-BART3-3p
                         |||****
5' UUUACCAUCAAUCAUCACCCUGUCCACCAACACUU 3'
```

OAS2 3'UTR

mut miR-BART1-3p

```
5' AUAACUCUGUGAUCUUGCUCUCGGUGCUUCCAAC  3'
                       |||||||
    3' CUGUAUCACCUAUCGCCACGAU 5'  miR-BART1-3p
                       |****||
5' AUAACUCUGUGAUCUUGCUCUCCCACCUUCCAAC 3'
```

JAK2 3'UTR

mut miR-BART2-5p

```
5' ACUUGCUGUUUUGAUUAAAAAGAAAAUAGUUUC  3'
                      ||||||||
   3' CGUUCCCGCUUACGUCUUUUAU 5'  miR-BART2-5p
                      |****|||
5' ACUUGCUGUUUUGAUUAAAAACUUUAUAGUUUC 3'
```

MAP3K2 3'UTR

mut miR-BART3-3p

```
5' UUUUGAAAGAUUGUGGUGCAAAGGCUUACUCUAA  3'
                 |||||||
3' UGUGGACCACUGAUCACCACGC 5'  miR-BART3-3p
                 ||****|
5' UUUUGAAAGAUUGUCCACCAAAGGCUUACUCUAA 3'
```

mut miR-BART22

```
5' UAUAUUGGCAUAUUGUGAUUCUUUGUAAUUGACU  3'
                      ||||||||
   3' UGAUGAUCUGGUACUGAAACAUU 5'  miR-BART22
                      ||****||
5' UAUAUUGGCUUAUUGUGAUUCUAACAAAUUGACU 3'
```

Supplementary Figure 2
